# Effect of Er: YAG laser on the bond strength of glass ceramic to bleached enamel and mechanism investigation

**DOI:** 10.1186/s12903-026-09745-x

**Published:** 2026-08-29

**Authors:** Jingwen Miao, Jing Ye, Junjie Wu, JiaHao Qin, Ben Ma, Jian Zhang, Ying Liu

**Affiliations:** 1 School and Hospital of Stomatology, Tianjin Medical University & Tianjin Key Laboratory of Oral Soft and Hard Tissues Restoration and Regeneration, Tianjin, 300070 China; 2 Department of Stomatology, Tianjin Hospital, Tianjin, 300211 China; 3 School and Hospital of Stomatology, Tianjin Medical University, Tianjin, 300070 China; 4 Department of Endodontics, Tianjin Binhai New Area Stomatologyical Hospital, Tianjin, 300450 China

**Keywords:** Tooth bleaching, Er:YAG laser, Glass-ceramic bonding, Degree of conversion, Shear bond strength

## Abstract

**Background:**

This study aimed to evaluate the efficacy of Er: YAG laser treatment for glass-ceramic bonding to bleached enamel and to further investigate the potential mechanisms.

**Methods:**

This in vitro study included 132 molars, with each molar used to prepare a single specimen. A total of 48 enamel slices, 48 enamel blocks, and 36 embedded whole-tooth specimens were prepared. The specimens were randomly allocated to four groups: the Control group, hydrogen peroxide (HP) group, HP combined with sodium ascorbate (HP-SA) group, and HP combined with Er: YAG laser (HP-Er: YAG) group. They were subsequently evaluated for enamel surface morphology, residual hydrogen peroxide (H_2_O_2_) concentration, resin degree of conversion (DC), resin penetration, shear bond strength (SBS), and failure mode.

**Results:**

SEM showed that the HP-Er: YAG group exhibited rough and irregular enamel surfaces, while the HP and HP-SA groups showed no obvious morphological changes. Compared with the HP group (109.60 ± 10.38 µmol/L), H_2_O_2_ concentrations were significantly lower in the HP-SA (51.05 ± 20.06 µmol/L) and HP-Er: YAG (29.96 ± 13.01 µmol/L) groups (*P* < 0.0001). The HP-Er: YAG group showed a lower concentration than the HP-SA group (*P* < 0.05).The DC of the HP-Er: YAG group (51.10 ± 7.02%) was comparable to that of the Control (59.84 ± 5.36%) and HP-SA (41.82 ± 7.28%) groups (*P* > 0.05). The Control and HP-Er: YAG groups exhibited extensive resin penetration at the enamel-adhesive interface. SBS was significantly higher in the Control (21.92 ± 4.33 MPa) and HP-Er: YAG (22.62 ± 4.06 MPa) groups than in the HP (13.46 ± 3.62 MPa) and HP-SA (17.76 ± 2.56 MPa) groups (*P* < 0.01). For ARI scores, the Control and HP-Er: YAG groups did not differ significantly (*P* > 0.05), whereas all other pairwise comparisons were statistically significant (*P* < 0.05).

**Conclusion:**

Reduced H_2_O_2_ levels, increased resin polymerization, and modification of the enamel surface may contribute to the improved bonding performance observed after Er: YAG laser treatment of bleached enamel. Compared with 10% SA treatment for 10 min, Er: YAG laser was more effective in restoring bond strength, suggesting its potential for immediate glass‑ceramic bonding to bleached enamel.

## Introduction

Tooth discoloration is a common esthetic concern in dentistry, and the demand for treatments that improve the appearance of discolored teeth has increased considerably in recent years. Consequently, veneers have become a widely used restorative approach [1, 2]. However, the final esthetic outcome of ceramic laminate veneers, particularly no-prep veneers, is influenced by the color of the underlying tooth structure. Because of their limited thickness, luting agents alone may not adequately mask the substrate color [3]. Therefore, dental bleaching is often performed before veneer cementation [3, 4]. The bleaching effect occurs through an oxidation process in which peroxide decomposition releases free radicals that penetrate the enamel and degrade pigment molecules into smaller, colorless compounds [5, 6]. Meanwhile, the widespread adoption of chairside CAD/CAM technology has enabled high-quality veneers to be fabricated and bonded in a single visit. This approach shortens treatment time, minimizes patient visits, and enhances patient comfort [7].

However, previous studies have shown that bleaching may reduce the bond strength of composite resin to enamel [6, 8]. Residual reactive oxygen species (ROS) generated from peroxide decomposition interfere with resin monomer infiltration and inhibit polymerization, potentially leading to premature termination of polymer chains [4, 8, 9]. In addition, bleaching has been reported to induce morphological and compositional changes in enamel, including increased porosity and surface roughness, disruption of the prismatic structure, and reduced calcium content [10, 11]. These alterations may weaken the mechanical properties of enamel and compromise adhesion during immediate cementation after tooth bleaching.

Several strategies have been suggested to mitigate the adverse effects of bleaching on the bond strength of composite resin restorations, among which delayed bonding is the most commonly recommended approach. The optimal waiting period remains uncertain, with reported intervals ranging from 24 h to 3 weeks to allow bond strength recovery [12]. However, the approach of delaying bonding may not always be practical for patients with limited treatment time [13]. Another commonly investigated approach is the use of antioxidants to neutralize residual oxidizing species and reduce their adverse effects on the bonding process [14, 15]. Among these agents, sodium ascorbate (SA), a reducing antioxidant, has received considerable attention because it has been reported to improve shear bond strength (SBS) after bleaching and may reduce the need for a waiting period before bonding in vitro [14, 16]. Nevertheless, there was considerable variation among studies regarding SA antioxidant treatment protocols for bleached enamel, particularly in terms of SA concentration, formulation, application technique, and treatment duration [17, 18]. Additionally, concerns regarding its potential biological effects and limited shelf life may restrict its clinical application [19, 20]. Therefore, alternative approaches that can effectively improve SBS while minimizing the adverse effects of bleaching are still needed.

Laser irradiation has been proposed as a promising method for improving the bonding performance of bleached enamel. In particular, the erbium-doped yttrium aluminum garnet (Er: YAG) laser may accelerate oxygen release, modify enamel surface morphology, and improve the SBS of bleached enamel post-bleaching SBS [21]. With a wavelength of 2940 nm, the Er: YAG laser is highly absorbed by water in mineralized tissues and has been shown to create a favorable substrate for subsequent restorative procedures [18].

However, studies investigating the mechanisms underlying glass-ceramic–enamel bonding after tooth bleaching remain limited. Therefore, the primary aim of this study was to evaluate the effect of Er: YAG laser treatment on residual hydrogen peroxide (H_2_O_2_) concentration, resin degree of conversion (DC), resin penetration, and the SBS of ceramic materials to bleached enamel. A secondary aim was to assess the potential of Er: YAG laser pretreatment for immediate glass‑ceramic bonding to bleached enamel.

## Materials and methods

### Specimen selection

The study was approved by the ethics committee of the Stomatological Hospital, Tianjin Medical University (TMUSHhMEC2020111). Written informed consent was obtained from all participants before specimen collection. A total of 132 human molars free of caries, cracks or hypoplastic lesions were collected. Soft tissue was removed from the molars using a periodontal scaler. Then, the teeth were stored in 1% thymol solution at 4 °C for 1 week. Before specimen preparation and subsequent analyses, the teeth were thoroughly rinsed with normal saline.

### Specimen preparation

Each tooth was prepared as a single specimen.

Preparation of 36 embedded whole-tooth specimens: The mesial and distal surface of the 36 molars were polished with 600-grit SiC paper under water cooling to create a flatten area, followed by ultrasonically cleaned for 10 min to remove debris. Each surface was examined under stereoscopic microscope to confirm that no dentin was exposed. The molars were then embeded perpendicularly in autopolymerizing acrylic resin up to cement-enamel junction within a hollow rectangular mold (10 mm×10 mm in cross section and 15 mm in height).

Preparation of 48 enamel slices: The mesial or distal enamel surfaces of the 48 crowns were sectioned to obtain enamel slices (3 mm × 3 mm × 1 mm) (Fig. 2A). The enamel surfaces were polished with 600-grit SiC paper and ultrasonically cleaned for 10 min.

Preparation of 48 enamel blocks: The 48 crowns were sectioned longitudinally in the buccolingual direction to obtain mesial or distal tooth halves (Figs. 2B and 3A). The enamel surfaces were polished with 600-grit SiC paper and ultrasonically cleaned for 10 min.

### Group allocation

Following specimen preparation for each analysis, the specimens were randomly allocated to four experimental groups using a computer-generated random sequence. The sequence was generated by an independent investigator who was not involved in specimen treatment or outcome assessment, and the order of testing was also randomized. The four groups were defined according to the enamel surface treatment: the Control group, the HP group, the HP-SA group, and the HP-Er: YAG group. The protocol for each group was as follows:

- Control group: Specimens in this group received no bleaching or surface conditioning.
- HP group: Specimens in this group were bleached but received no further treatment. The surfaces of samples were bleached using 37.5% hydrogen peroxide (HP; SDI Limited, Bayswater, Victoria, Australia) according to the manufacturer’s instructions. A 1 mm thick layer of bleaching gel was applied to an approximately 3 mm × 3 mm area of the enamel surface and left in place for 8 min. The gel was then removed, and the surface was rinsed with water for 2 min and air-dried. The procedure was repeated twice.
- HP-SA group: Bleaching was first performed according the same protocol used for the HP group. Subsequently, a 10% SA solution (Solarbio, Beijing, China) was applied to the bleached surface with a brush for 10 min, with the surface kept moist throughout the treatment. The surface was rinsed with water for 2 min and air-dried.
- HP-Er: YAG group: Bleaching was first performed according the same protocol used for the HP group. The specimens were then irradiated with an Er: YAG laser (Fotona, Ljubljana, Slovenia) at 120 mJ /pulse, 10 Hz, and 1.2 W in non-contact mode with a 1-mm working distance. The laser was operated in MSP mode with a water/air spray ratio of 5/4. The enamel bonding area was uniformly irradiated using a sweeping motion for six irradiation cycles of 10 s each. The surfaces were rinsed with water for 2 min and air-dried.

### Morphological evaluation

Six enamel slices from each group were selected for observation of enamel surface morphology. The specimens were fixed in 2.5% glutaraldehyde solution (Solarbio, Beijing, China) for 4 h, rinsed twice with 0.1 mol/L PBS (Solarbio, Beijing, China), and dehydrated through a graded ethanol series (50%, 75%, and 90% for 15 min each, followed by two changes of 100% ethanol for 30 min each). The specimens were then vacuum dried and sputter coated with gold before examination by scanning electron microscopy (SEM; FEI, Hillsboro, OR, USA).

### H_2_O_2_ detection

Six enamel slices from each group were immediately selected for H_2_O_2_ detection using a method described in a previous study [22]. Each slice was individually immersed in 10 mL of deionized water and stored at 4 ℃ for 48 h. The solutions were then transferred to the initial reaction system (5.0 × 10^− 4^ mol/L FeSO_4_, 3.0 × 10^− 5^ mol/L methyl orange [MO], pH 2.5) (Shanghai Yien Chemical Technology Co., Ltd., Shanghai, China). After 2 min of reaction, the residual MO, represented by the absorbance value (A_2_), was measured at 507 nm using a UV–visible spectrophotometer (Mapada Instruments, Shanghai, China). For the initial absorbance (A_1_), deionized water was added to the same reaction system and allowed to react for 2 min under identical conditions, after which the absorbance was measured at 507 nm. The H_2_O_2_ concentration was calculated using the following equation:

$$\triangle\mathrm{A}=0.008\mathrm{C}\:_{\mathrm{H}_2\mathrm{O}_2}+0.0284$$

$$\triangle\mathrm{A}:\mathrm{A}_1-\mathrm{A}_2$$

$$\mathrm{C}\:_{\mathrm{H}_2\mathrm{O}_2}:\mathrm{H}_2\mathrm{O}_2\:\mathrm{concentration}$$

### DC of the resin adhesive measurement

Six enamel blocks from each group were immediately selected to evaluate the DC of the resin adhesive. The specimens were etched with 35% phosphoric acid (Heraeus Kulzer, Hanau, Germany) for 30 s, rinsed with water for 30 s, and then air-dried. Single Bond Universal (3 M ESPE, St. Paul, MN, USA) was applied to the etched surface according to the manufacturer’s instructions and light-cured for 20 s. The specimens were subsequently sectioned longitudinally across the bonded interface to obtain 1 mm thick slices. These slices were polished with 1000, 1500, 2000, 2500 and 3000-grit SiC paper and ultrasonically cleaned for 10 min. The slices were then stored in deionized water for 24 h before testing.

Raman spectra were acquired using a Raman Microscope (Thermo Fisher Scientific, Waltham, USA). The micro-Raman parameters were performed using a 532-nm laser with a power of 30 mW, a spectral resolution of 5.8–8.8 μm, and a spot size of approximately 1.1 μm. Measurements were obtained from the adhesive layer adjacent to the treated enamel surface. A digital image of each measurement site was recorded before spectral acquisition. Spectral data were processed using LabSpec 5.0 software.

The DC of the resin adhesive was calculated from the ratio of the peak intensities at 1639 cm^− 1^ and 1609 cm^− 1^ before and after curing. The spectrum of the uncured adhesive was used to determine the baseline ratio before polymerization, according to the following equation:

$$\begin{aligned}\mathrm{DC}&=\left[1\:\mathrm{-}\:\left(1639\:\mathrm{cm}^{-1}/\:1609\:\mathrm{cm}^{-1}\right)\mathrm{peak}\:\mathrm{height}\:\mathrm{after}\:\mathrm{curing}/\left(1639\mathrm{cm}^{-1}/\:1609\:\mathrm{cm}^{-1}\right)\mathrm{peak}\:\mathrm{height}\:\mathrm{before}\:\mathrm{curing}\right]\times100\%\end{aligned}$$

### Resin penetration observation

Six enamel blocks from each group were immediately selected for confocal laser scanning microscopy (CLSM; Carl Zeiss, Oberkochen, Germany) to evaluate resin penetration. The specimens were etched with 35% phosphoric acid for 30 s, rinsed with water for 30 s, and air-dried. Single Bond Universal adhesive was mixed with Rhodamine B (Shanghai Yien Chemical Technology Co., Ltd., Shanghai, China) to a final concentration of 0.1 wt% and applied to the etched enamel surface according to the manufacturer’s instructions. After light curing for 20 s, all specimens were sectioned longitudinally across the bond surface to obtain 1 mm thick slices. These slices were sequentially polished with 1000, 1500, 2000, 2500 and 3000-grit SiC paper, ultrasonically cleaned for 10 min, and examined using CLSM. Images were obtained using an excitation wavelength of 568 nm.

### Shear bond strength testing

IPS e.max CAD lithium disilicate glass-ceramic ingots (Ivoclar Vivadent, Schaan, Liechtenstein) were used to prepare 72 standardized ceramic specimens, each measuring 3 mm in diameter and 6 mm in height.

All ceramic specimens were bonded to the mesial and distal enamel surfaces of the molars by the same experienced clinician. The flattened enamel surfaces of the molars in each group were immediately etched with 35% phosphoric acid for 30 s, rinsed with water for 30 s, and air-dried. Single Bond Universal was then applied to the etched surface according to the manufacturer’s instructions and light cured for 20 s. Meanwhile, the bonding surfaces of the ceramic specimens were etched with 5% hydrofluoric acid (Ivoclar Vivadent, Schaan, Liechtenstein) for 20 s and rinsed with water for 20 s to remove residual acid. After thorough air-drying, the silane (Porcelain Primer; Bisco, Schaumburg, IL, USA) was applied to the ceramic surface for 30 s according to the manufacturer’s instructions and then gently air dried for 10 s. Single Bond Universal was subsequently applied to the ceramic surface according to the manufacturer’s instructions. Rely™ Ultimate cement (3 M ESPE, St. Paul, MN, USA) was applied to the ceramic surface, and each ceramic specimen was seated onto the treated tooth surface. Most of the excess resin cement was removed before light curing, followed by light curing from the buccal and lingual surfaces for 40 s each. Any remaining excess cement was then carefully removed with a blade after light curing. Finally, the bonded specimens were stored in a 100% humid environment for 24 h before the SBS test.

The SBS was measured using a universal test machine (Instron, Norwood, MA, USA) at a crosshead speed of 1 mm/min. The specimens were positioned so that the bonded ceramic block was perpendicular to the direction of the applied shear load. A knife-edge plunger was positioned 1 mm above the bonding interface to generate the shear force. The maximum load at failure was recorded at debonding, and SBS was calculated as the fracture load divided by the bonding area.

$$\begin{aligned}\mathrm{SBS}\:\left(\mathrm{MP}_\mathrm{a}\right)&=\mathrm{Maximum}\:\mathrm{load}\:\mathrm{at}\:\mathrm{failure}\:\left(\mathrm{N}\right)\\&/\:\mathrm{Cross-sectional}\:\mathrm{bonding}\:\mathrm{area}\:\left(\mathrm{mm}^2\right)\end{aligned}$$

### Failure mode

After the SBS test, the molars were examined under a stereomicroscope (Leica Microsystems, Wetzlar, Germany) at 30× magnifications to assess the failure mode. The adhesive remnant index (ARI) was used to evaluate the amount of adhesive remaining on the enamel surface. The ARI scores were assigned on a 0–3 scale according to the criteria described in a previous study [23].

- 0: No adhesive remaining on the enamel surface.
- 1: Less than 50% of the adhesive remaining on the enamel surface.
- 2: More than 50% the adhesive remaining on the enamel surface.
- 3: All adhesive remaining on the enamel surface.

### Statistical analysis

Statistical analyses were performed using SPSS version 26.0 (IBM Corp., Armonk, NY, USA). Normality was assessed using the Shapiro–Wilk test, together with inspection of Q–Q plots and individual data distributions, whilehomogeneity of variance was evaluated using Levene’s test. Levene’s test indicated homogeneity of variance for DC, H_2_O_2_, and SBS data (all *P* > 0.05). The Shapiro–Wilk test showed no significant departure from normality for DC and H_2_O_2_ data (all *P* > 0.05). For SBS, the Control group showed a significant departure from normality (*P* = 0.009), whereas no significant departure was detected in the other three groups (all *P* > 0.05). Given the balanced sample sizes (*n* = 18 per group), homogeneous variances, and the robustness of one-way ANOVA to moderate departures from normality, one-way ANOVA was retained for the SBS analysis. One-way ANOVA was also used for DC and H_2_O_2_ data. Tukey’s HSD test was used for post hoc pairwise comparisons of DC and H_2_O_2_, whereas Bonferroni-adjusted pairwise comparisons were applied for SBS. ARI scores, as ordinal data, were compared among groups using the Kruskal–Wallis test, followed by pairwise Mann–Whitney U tests with adjustment for multiple comparisons. A *p*-value of less than 0.05 was considered statistically significant (*P* < 0.05).

## Results

### Morphological evaluation

**Fig. 1:**
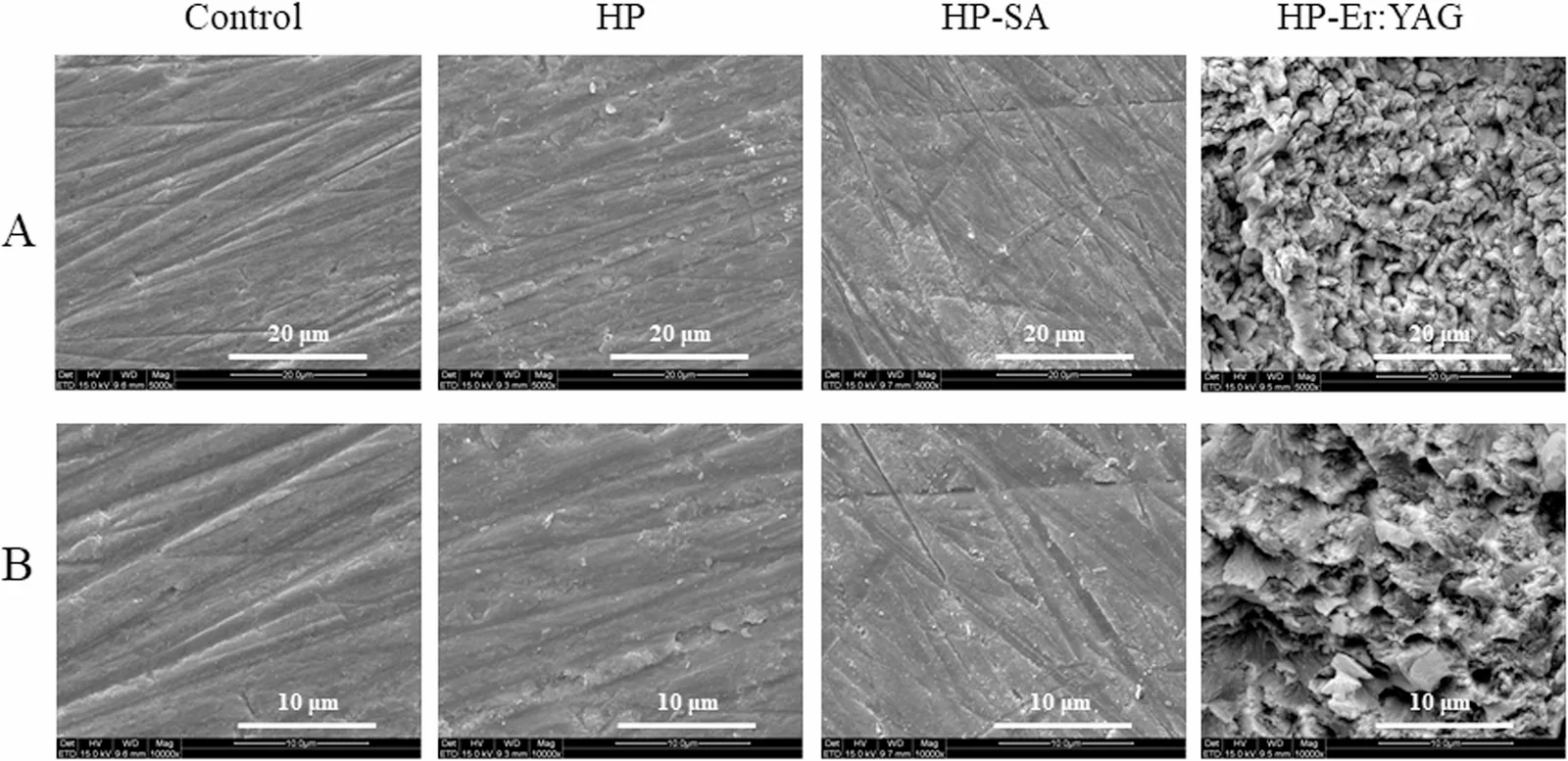
SEM images of enamel surface morphology in the four groups. **A** Scale bar, 20 μm; **B** scale bar, 10 μm

SEM analysis revealed distinct changes in enamel surface morphology after Er: YAG laser irradiation. Across the six specimens examined in the HP-Er: YAG group, the enamel surface appeared markedly rough and irregular, with crater-like pits of varying sizes and uneven contours. In contrast, no obvious morphological changes were observed in the HP or HP-SA groups, and their enamel surfaces appeared similar to those of the Control group, with an overall smooth and intact appearance and only scratches of varying depths observed. Representative images illustrating these characteristic patterns are shown in Fig. 1.

### H_2_O_2_ detection

**Fig. 2:**
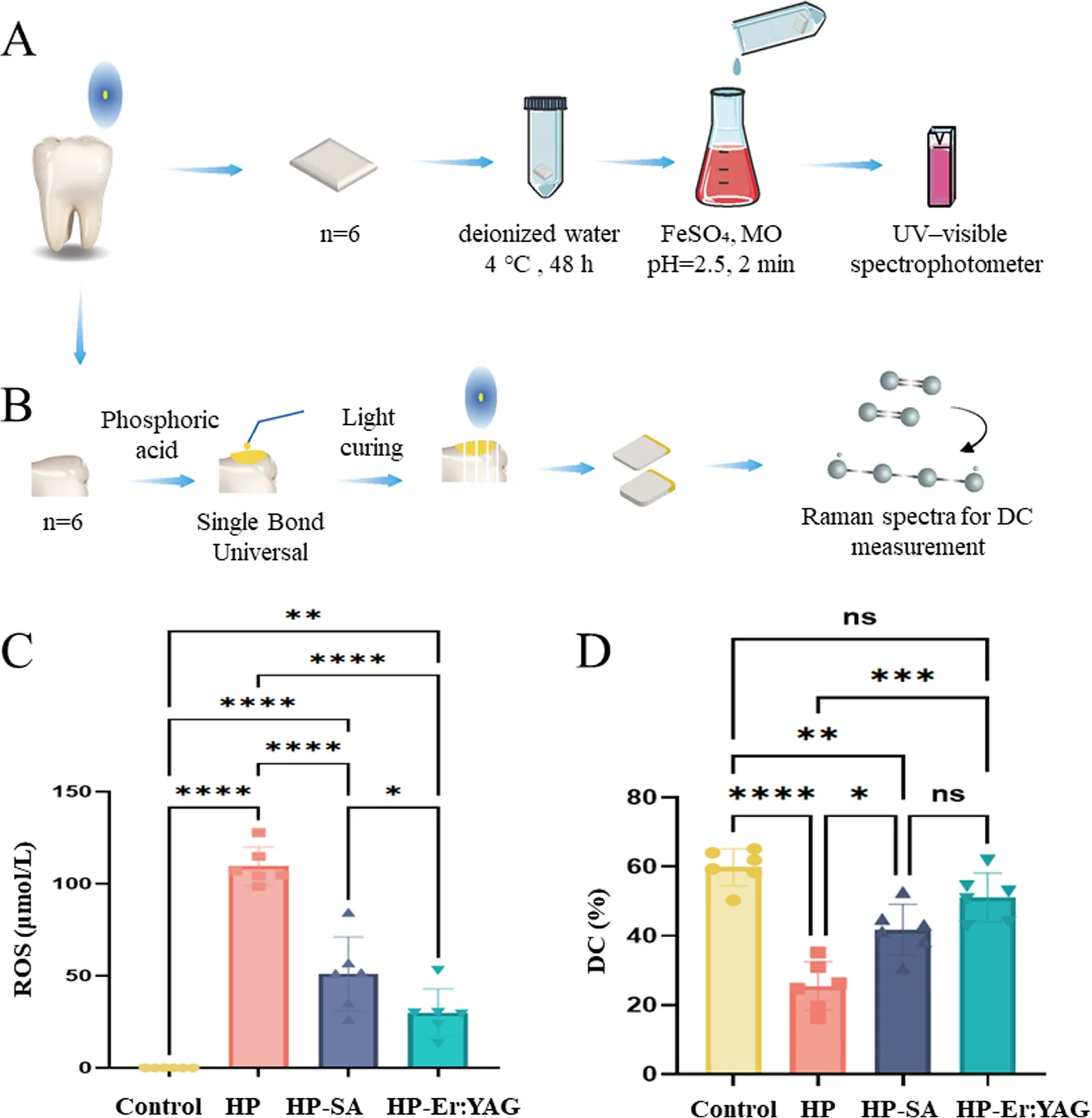
H_2_O_2_ detection and DC measurement results across four groups. **A** Schematic illustration of the H_2_O_2_ detection procedure; **B** Schematic illustration of the DC measurement procedure; **C** H_2_O_2_ concentrations; **D** DC values (*P < 0.05, **P < 0.01, ****P < 0.0001)

H_2_O_2_ concentration was detected in the enamel surfaces of all three bleached groups, whereas almost no detectable H_2_O_2_ was observed in the Control group (Fig. 2C). Compared with HP group (109.6 ± 10.38 µmol/L), both HP-SA (51.05 ± 20.06 µmol/L) and HP-Er: YAG (29.96 ± 13.01 µmol/L) groups exhibited significantly lower H_2_O_2_ concentrations (*P* < 0.0001). In addition, the H_2_O_2_ concentration was higher in the HP-SA group than that in the HP-Er: YAG group (*P* < 0.05).

### DC measurement

The DC of resin adhesives polymerized on the pretreated enamel surface is shown in Fig. 2D. Among the four groups, the HP group showed the lowest DC value (25.47 ± 7.03%). The Control group exhibited a significantly higher DC value (59.84 ± 5.36%) than the HP group (*P* < 0.0001) and the HP-SA group (41.82 ± 7.28%, *P* < 0.01). In addition, no statistically significant differences were observed between the Control and HP-Er: YAG groups (51.10 ± 7.02%) or between the HP-SA and HP-Er: YAG groups (*P* > 0.05).

### Resin penetration observation

**Fig. 3:**
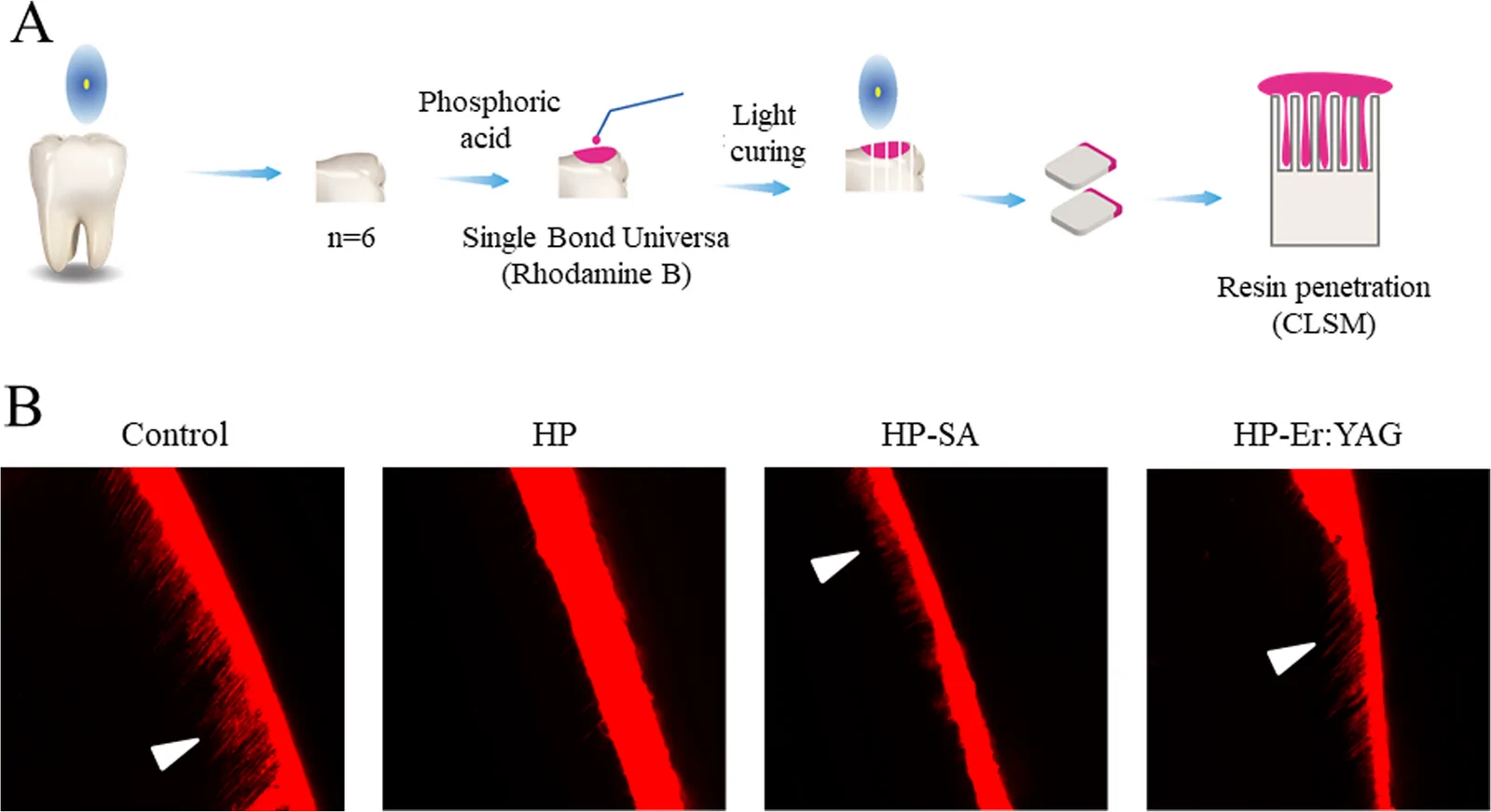
Observation of resin penetration across four groups. **A** Schematic illustration of the resin penetration procedure; **B** CLSM images illustrating resin penetration into the enamel substrate

CLSM was used to qualitatively visualize resin penetration at the enamel–adhesive interface. Across the six specimens examined in each group, the Control and HP-Er: YAG groups generally showed extensive resin penetration, whereas resin penetration appeared less extensive in the HP-SA group. In the HP group, resin penetration was rarely observed. Representative images illustrating these characteristic patterns are shown in Fig. 3B.

### SBS testing

**Fig. 4:**
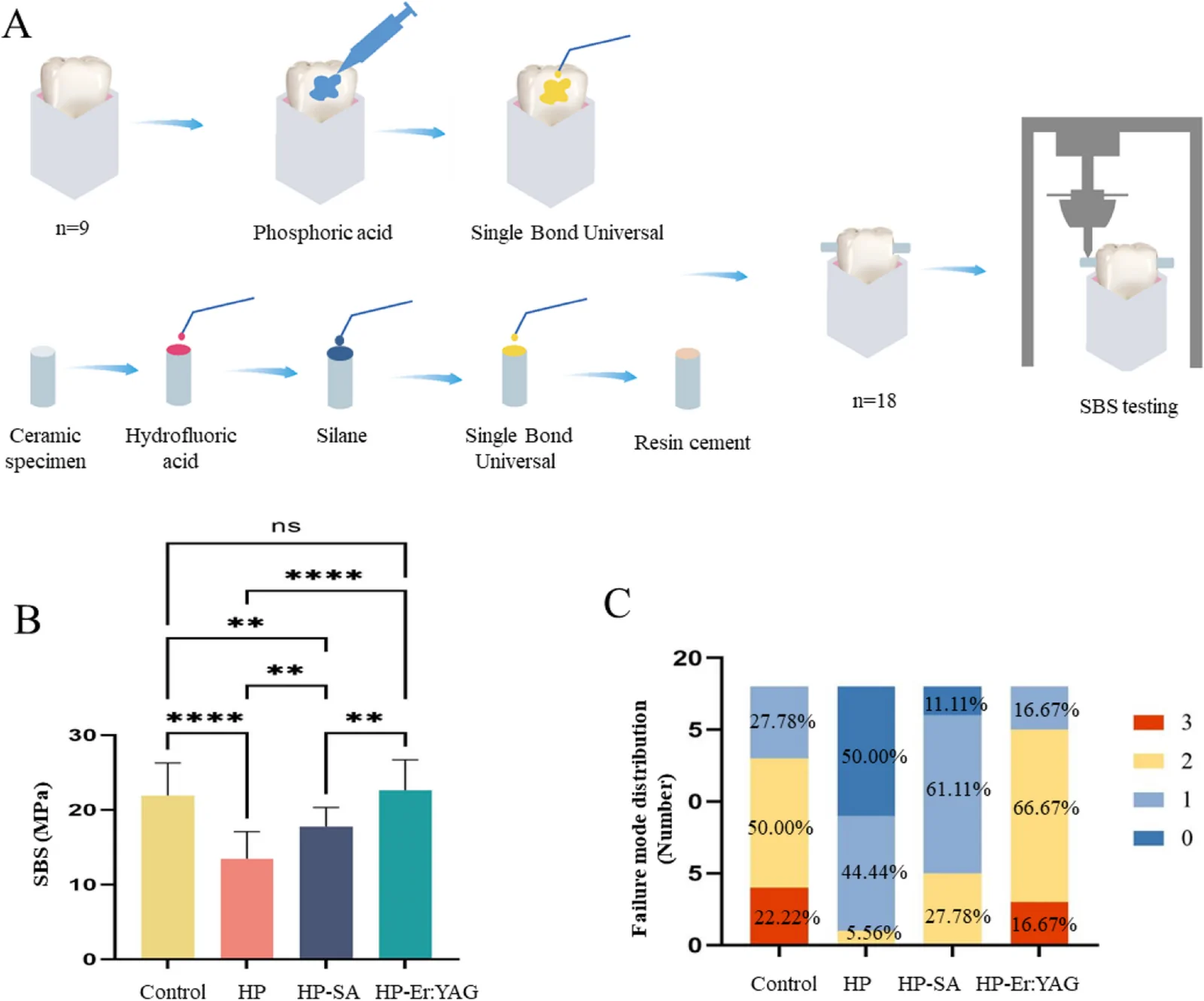
SBS testing and failure modes across the four groups. **A** Schematic illustration of the experimental procedure; **B** SBS results; **C **ARI scores (**P < 0.01, ****P < 0.0001)

The mean SBS values and standard deviations for each group are presented in Fig. 4B. No significant difference was observed between the Control group (21.92 ± 4.33 MPa) and the HP-Er: YAG group (22.62 ± 4.06 MPa). Both groups exhibited significantly higher SBS values than the HP group (13.46 ± 3.62 MPa) and HP-SA group (17.76 ± 2.56 MPa) (*P* < 0.01). The HP group showed significantly lower SBS than all other groups (*P* < 0.01).

### Failure mode

The distribution of ARI scores is presented in Fig. 4C. Score 2 was the most prevalent in the Control and HP-Er: YAG groups, indicating that more than half of the adhesive remained on the enamel surface. In comparison, the HP and HP-SA groups showed a higher proportion of scores 0 and 1, indicating less adhesive residue on the enamel surface. No statistically significant difference was observed between the Control and HP-Er: YAG groups, whereas statistically significant differences were found between all other pairwise group comparisons (*P* < 0.05).

## Discussion

Residual oxidative substances generated during bleaching can impair the immediate bonding of enamel and compromise the long-term stability of adhesive restorations. Consistent with previous studies, bleaching adversely affects the immediate bond strength between restorative materials and enamel [6, 18, 24]. Therefore, effective approaches for restoring the bonding performance of bleached enamel before glass-ceramic restoration remain to be clarified. In the present study, we compared the effects of Er: YAG laser and 10% SA treatments on the bonding performance of bleached enamel. In addition, the potential mechanisms, particularly residual H_2_O_2_ and resin polymerization, were investigated. By integrating bond strength and mechanistic analyses, this study provides insight into impaired glass-ceramic bonding to bleached enamel.

Consistent with previous findings, our results showed that HP bleaching markedly impaired the bonding between enamel and glass ceramic. A SA, a commonly used antioxidant, may improve the bonding performance of bleached enamel by neutralizing residual oxidizing species through electron donation and restoring the local redox balance [14, 15]. However, 10% SA treatment for 10 min only partially alleviated the bleaching-induced reduction in bond strength and could not restore SBS or ARI scores to the levels observed in unbleached enamel. This finding is consistent with previous studies showing that SA treatment does not completely recover the bond strength of bleached enamel [16, 25]. Dabas et al. reported that SBS increased with longer application times of SA hydrogel (30, 60, and 120 min), indicating that prolonged exposure may further improve the bond of composite resin to bleached enamel [26]. However, longer treatment time may reduce clinical efficiency and practicality in routine restorative procedures [6]. In contrast, Er: YAG laser treatment showed a greater improvement in bonding performance under the conditions tested. The HP-Er: YAG group exhibited SBS and ARI scores comparable to those of the Control group, while the laser treatment required less treatment time and involved a simpler procedure than SA treatment. These findings suggest that Er: YAG laser treatment may be a promising approach for improving the bonding performance of bleached enamel.

Tooth bleaching can be performed using either at-home or in-office techniques, with some patients choosing the higher-concentration in-office approach to avoid wearing trays daily for several weeks [27]. Therefore, an in-office bleaching protocol using 37.5% HP gel was employed in the present study before bonding the ceramic specimens to enamel. During bleaching, highly reactive molecules initiate oxidative reactions that interact with and break down stain molecules within the enamel substrate, resulting in a whitening effect [28, 29]. Cakir et al. reported the alterations in the chemical composition of enamel after bleaching, including an increase in oxygen content [30]. Unlike prior studies that only speculated on oxygen free radical induced bonding impairment, the present study quantitatively detected H_2_O_2_ concentration on bleached enamel surfaces using the oxidation induced decolorization of MO in a Fenton reaction system. As an azo dye, MO can be efficiently decolorized in a Fenton system, primarily through the addition of ^•^OH radicals to the double bonds of its azo group [22]. The degree of MO decolorization, measured at 507 nm, is directly related to the H_2_O_2_ concentration, providing a relatively simple, rapid, and sensitive spectrophotometric approach for H_2_O_2_ detection. In this study, H_2_O_2_ concentration was detected in the enamel surfaces from all bleached groups. The HP group showed the highest H_2_O_2_ concentration, whereas both the HP-SA and HP-Er: YAG groups significantly reduced the residual H₂O₂ level. Notably, the reduction was greater following Er: YAG laser irradiation than after SA treatment. The different effects of these two treatments may be related to their distinct mechanisms of action. SA exerts its antioxidant effect mainly through rapid redox reactions occurring at the outermost enamel surface, with most antioxidant activity taking place during the initial minutes of exposure [6]. In addition, the high fluidity of the SA solution, together with its concentration and application time, may collectively limit its ability to neutralize residual H_2_O_2_ [25, 31]. In comparison, the photomechanical effect produced by Er: YAG laser irradiation enables enamel preparation at varying depths. Depending on the irradiation parameters, including energy, frequency, and exposure time, laser treatment may facilitate the release of residual oxidizing species while simultaneously forming uniform micro-ablation structures on the enamel surface [18, 21, 32]. This dual function of reducing residual H_2_O_2_ and modifying the enamel surface may therefore contribute to the improved bonding performance observed after Er: YAG laser treatment.

Excessive residual H_2_O_2_ within the enamel structure may interfere with resin monomer infiltration into the interprismatic regions and leads to premature chain termination and incomplete polymerization [33]. Unreacted monomer within the hybrid layer may increase the permeability of the adhesive interface, resulting in a porous and poorly sealed structure that is more susceptible to degradation [34]. The DC directly reflects the extent of resin polymerization and is closely related to the mechanical properties of the adhesive interface. Inadequate polymerization, as indicated by a low DC, may compromise the mechanical performance of adhesive materials and performance [35, 36]. Following the methodology described by Hass et al. [34]., micro-Raman spectroscopy was used to analyze the DC of the adhesive layer o adjacent to the treated enamel surface. In the present study, DC measurements demonstrated that HP bleaching significantly decreased the DC of the resin adhesive. Both the HP-SA and HP-Er: YAG groups improved resin polymerization compared with the HP group. Notably, the DC of the HP-Er: YAG group did not differ significantly from that of the Control group. These findings provide a potential mechanistic explanation for the effects of different treatments on the bonding performance of bleached enamel, although the direct relationship between DC and bond strength requires further investigation.

SEM and CLSM were used for qualitative evaluation of enamel surface morphology and resin penetration, respectively. SEM images showed that conventional HP bleaching did not cause obvious morphological changes to the enamel surface. The differences in bleached enamel morphology reported among previous studies may be related to variations in bleaching agent composition, peroxide concentration, pH, manufacturers’ protocols, application duration, and bleaching techniques [25, 28]. In our SEM study, Er: YAG laser treatment produced a distinctly rough and uneven enamel surface. Erbium lasers modify dental hard tissues through photothermal and photomechanical effects. The absorption of laser energy by water can induce rapid vaporization and micro-explosive effects, resulting in surface micro-irregularities, while appropriate irradiation parameters may help minimize excessive thermal effects on the surrounding tissues [21, 37, 38]. Consistent with our observations, Akhoundi et al. reported that Er: YAG laser irradiation at 200 mJ and 10 Hz created irregular and amorphous enamel surfaces under SEM observation, with the resulting surface irregularities potentially contributing to the mechanical retention of bonding materials [39]. Unlike dehydrated SEM observation, CLSM allows nondestructive observation of specimens under hydrated conditions, providing clear visualization of resin penetration and the enamel–adhesive interface with fewer artifacts [40, 41]. In this study, the HP-Er: YAG group exhibited extensive resin penetration, with a morphology comparable to that observed in the Control group. In contrast, the HP-SA group showed sparse and relatively limited resin penetration. This difference may be associated with residual H_2_O_2_ and the limited morphological modification of the enamel surface by SA treatment. In the HP group, resin penetration was rarely observed, which may be related to the higher residual H_2_O_2_ level and the reduced polymerization observed in this group.

This study has several limitations that need to be acknowledged. All experiments were conducted under standardized in vitro conditions and therefore could not fully reproduce the clinical oral environment, including saliva exposure, thermal fluctuations, and cyclic occlusal loading. In addition, the SA protocol used in this study (10% SA for 10 min) was based on a previously reported protocol, and different antioxidant concentrations and application times were not systematically compared with Er: YAG laser treatment. Therefore, the present findings should not be interpreted as evidence that Er: YAG laser treatment is superior to sodium ascorbate under other treatment conditions. However, these findings can suggest that Er: YAG laser pretreatment may be a potential approach for immediate ceramic bonding after tooth bleaching. Further clinical studies are needed to confirm these findings and optimize the treatment parameters for immediate ceramic bonding following tooth bleaching.

## Conclusion

In summary, this study suggested that residual H_2_O_2_ accumulation may adversely affect resin polymerization and penetration into enamel, which may collectively contribute to reduced bond strength between glass ceramic and enamel. Er: YAG laser pretreatment may improve the bonding performance of bleached enamel by reducing residual H_2_O_2_, promoting resin polymerization, and modifying the enamel surface morphology. Under the tested conditions, Er: YAG laser treatment showed greater bonding efficacy than 10% SA treatment for 10 min. These findings suggest that Er: YAG laser pretreatment may have potential for immediate glass-ceramic bonding after tooth bleaching.

## Data Availability

The datasets used and/or analyzed during the current study are available from the corresponding author on reasonable request.

## Abbreviations

PBS: Phosphate buffered saline
min: Minute
h: Hour
s: Second
ml: Milliliter
μL: Microliter
μmol: Micromole
mol: Mole
mm: Millimeter
nm: Nanometer
N: Newton
MPa: Megapascal
SPSS: Statistical Package for the Social Sciences
ANOVA: Analysis of Variance
